# Identification of Epithelial–Mesenchymal Transition-Related lncRNA With Prognosis and Molecular Subtypes in Clear Cell Renal Cell Carcinoma

**DOI:** 10.3389/fonc.2020.591254

**Published:** 2020-11-25

**Authors:** Weimin Zhong, Fengling Zhang, Chaoqun Huang, Yao Lin, Jiyi Huang

**Affiliations:** ^1^ Central Laboratory, The Fifth Hospital of Xiamen, Xiamen, China; ^2^ Traditional Chinese Medicine Department, The Fifth Hospital of Xiamen, Xiamen, China; ^3^ Key Laboratory of Optoelectronic Science and Technology for Medicine of Ministry of Education, College of Life Sciences, Fujian Normal University, Fuzhou, China,; ^4^ Department of Nephrology, The Fifth Hospital of Xiamen, Xiamen, China

**Keywords:** clear cell renal cell carcinoma, epithelial–mesenchymal transition, lncRNA, prognostic model, nomogram, molecular subtype

## Abstract

Epithelial–mesenchymal transition (EMT), a reversible cellular program, is critically important in tumor progression and is regulated by a family of transcription factors, induction factors, and an array of signaling pathway genes. The prognostic role and biological functions of EMT-related lncRNAs in ccRCC are largely unknown. In the present study, we analyzed the gene expression data and clinical information retrieved from The Cancer Genome Atlas (TCGA) database (N=512) and International Cancer Genome Consortium (ICGC) database (N=90) which served as training and external validation dataset, respectively. Then, we constructed an EMT-related lncRNA risk signature based on the comprehensive analysis of the EMT-related lncRNA expression data and clinical information. The Kaplan-Meier curve analysis revealed that patients in the low-risk and high-risk groups exhibited significant divergence in the overall survival (OS) and disease-free survival (DFS) of ccRCC, as was confirmed in the validation dataset. The Cox regression analysis of the clinical factors and risk signature in the OS and DFS demonstrated that the risk signature can be utilized as an independent prognostic predictor. Moreover, we developed an individualized prognosis prediction model relying on the nomogram and receive operator curve (ROC) analysis based on the independent factors. The Gene Set Enrichment Analysis (GSEA) indicated that patients in the low-risk group were associated with adherens junction, focal adhesion, MAPK signaling pathway, pathways in cancer, and renal cell carcinoma pathway. In addition, we identified three robust subtypes (named C1, C2 and C3) of ccRCC with distinct clinical characteristics and prognostic role in the TCGA dataset and ICGC dataset. Among them, C1 was associated with a better survival outcome, whereas C2 and C3 was associated with a worse survival outcome and have more advanced-stage patients. Moreover, C2 was more likely to respond to immunotherapy and was sensitive to chemo drugs, this may provide insights to clinicians to develop an individualized treatment. Collectively, this work developed a reliable EMT-related lncRNA risk signature that can independently predict the OS and DFS of ccRCC. Besides, we identified three stable molecular subtypes based on the EMT-related lncRNA expression, which may comprehensively be vital in elucidating the underlying molecular mechanism of ccRCC.

## Introduction

Clear cell renal cell carcinoma (ccRCC) is the most common type of renal cell carcinoma, accounting for 70–80% of RCC cases (1). Surgical resection is the major treatment for localized ccRCC, however, it is associated with a poor prognosis, and approximately 30–40% of ccRCC patients develop to metastatic recurrence during the follow-up (2). Currently, immunotherapies such as programmed death-1 (PD-1)/programmed death-ligand 1 (PD-L1) or cytotoxic T-lymphocyte-associated protein 4 (CTLA-4) inhibitors have been approved for managing ccRCC (3). Also, such therapies have proved to inhibit immune checkpoints, thus show efficacy and achieve some progress. However, a percentage of patients remain response poorly, and developed to resistance or progression (4). Consequently, there is an urgent need to identify new biomarkers or therapeutic targets to predict ccRCC progression, prognosis, and response to treatment.

Epithelial–mesenchymal transition (EMT) refers to a biological process in which epithelial cells dedifferentiate to mesenchymal cells (5, 6). During the EMT process, the epithelial cells lose their polarity, cytoskeletal structure, and cell-cell adhesion but retrieved the migratory properties typical from mesenchymal cells (7). Moreover, a report showed that EMT linked to fibrosis exhibited a prominent feature in ccRCC, which is closely related to a worse survival outcome (8). Thus, the EMT-related genes may serve as a promising target for future therapeutic interventions. However, the expression profile of EMT-related lncRNA, the diverse pathological features of ccRCC and their prognostic value have not been systematically explored. EMT involves substantial molecular reprogramming of cells which endows several clinical features to cancer cells and significantly impact in tumor cell interactions within the tumor microenvironment (9). In addition, EMT is also known to play a crucial role in drug resistance, whereas massive EMT-associated pathways contribute to the drug resistance in tumor cells (10). The cells which are subjected to the EMT process exhibit similar functions to cancer stem cells (CSC), for example, increased drug efflux pumps and anti-apoptotic effects (10). Thus, targeting EMT is regarded as a potential treatment strategy to overcome drug resistance.

Herein, we analyzed the RNAseq data and corresponding clinical information retrieved from TCGA (N=512) and ICGC (N=90 ) database to comprehensively explore the prognostic role of EMT-related lncRNA in ccRCC. Moreover, we assessed the potential molecular subtypes of EMT-related lncRNA in ccRCC patients and analyzed the association between subtypes and immunotherapy, as well as drug sensitivity and tumor microenvironment.

## Materials and Methods

### Data Acquisition

The RNAseq reads count and clinical information were obtained from the TCGA database (https://portal.gdc.cancer.gov/) and ICGC database (https://dcc.icgc.org/), respectively. To ensure high-quality analyses, we retained the samples with a survival time of ≥30 days. Subsequently, 512 ccRCC patients from the TCGA and 90 ccRCC patients from the ICGC database were included in downstream analysis. Moreover, information on the disease-free survival was retrieved from the cbioportal database (https://www.cbioportal.org/). Moreover, the RNAseq transcriptome data were transformed in transcript per million (TPM) value.

### Correlation Analysis

Here, we downloaded 200 EMT-related genes from the Molecular Signature Database v7.1 (MSigDB) (http://www.broad.mit.edu/gsea/msigdb/). To identify the EMT-related lncRNAs, we firstly extracted all lncRNA expression data in the TCGA database based on the GENCODE project (http://www.gencodegenes.org). Then, Pearson correlation analysis was performed between EMT-related genes and all lncRNA expression data in samples to identify the EMT-related lncRNA on the basis of the correlation coefficient and p values (**|**Cor _pearson_ **| >** 0.3 and p value < 0.01).

### Constructing a Risk Signature

A univariate Cox regression analysis was performed to select significant EMT-related lncRNAs associated with the survival of ccRCC (p < 0.05). Then, we selected the lncRNAs with significant clinical variables and conducted on feature selection using the randomForestSRC software package (https://cran.r-project.org/package=randomForestSRC). The randomSurvivalForest algorithm was employed to rank the prognostic genes (ntree=1000) based on their importance. The lncRNAs with a relative importance > 0.4 were subjected to a multivariate Cox regression analysis, after which we constructed a risk signature using the Akaike information criterion for stepwise backward/forward model selection. The risk score for each patient was calculated using the risk formula:

(1) $$Riskscore=\sum_{i=1}^{N}\left(Exp{\;}_{i}\;*\;\beta {\;}_{i}\right),$$

where Exp _i_ represent the expression of each prognostic lncRNA and β _i_ represent the coefficient of each prognostic lncRNA.

### GSEA Enrichment Analysis

To identify the potential KEGG pathway that involved in the lncRNA risk signature, Gene Set Enrichment Analysis (GSEA) was performed to find the significant enriched term in the high-risk and low-risk group The pathways with p < 0.05 and FDR < 0.05 were considered as statistically significantly.

### Independence of the EMT-Related lncRNA Signature

The lncRNA signature and corresponding clinical information of the DFS and OS were exploited to identify the independence using the univariate Cox regression analysis and multivariate cox regression analysis. p < 0.05 was considered as statistically significant.

### Nomogram Construction and Validation

The nomogram was established based on the all independent prognostic factors using the rms R package (https://cran.r-project.org/web/packages/rms/index.html). The calibration plot curve analysis was applied to evaluate the discrimination and the calibration of the nomogram.

### Non-Negative Matrix Factorization (NMF) Clustering

To explore the potential molecular subtype, a non-negative matrix factorization (NMF) clustering algorithm was applied to cluster the ccRCC sample via the “NMF” R package (11). The number of cluster k was set from 2 to 7. Then, we selected the optimal k value based on the cophenetic coefficient. The gene mutation for each subtype was calculated, from which we selected the top 20 genes to visualized using the maftools R package.

### Tumor Microenvironment Analysis

To assess the tumor microenvironment in ccRCC, we determined the infiltration levels of 22 immune cells using the CIBERSORT algorithm based on the all gene expression levels. We uploaded the gene expression data the to the CIBERSORTx web portal. Then, the algorithm was run using the LM22 signature for 1000 permutations (12). The ccRCC samples with an output P-value < 0.05 were selected for further analysis. Moreover, the immune core, and the stromal score were calculated using the “estmate” R package (http://r-forge.r-project.org).

### Predicting Chemotherapeutic Response

We predicted the chemotherapeutic response for each ccRCC patient based on information retrieved from the Genomics of Drug Sensitivity in Cancer (GDSC) database (13). Two common chemo drugs, sorafenib and sunitinib, which have been approved for treating of metastatic RCC cases were selected to predict the chemotherapeutic response (14). The prediction procedure was conducted using the R package “pRRophetic” where the half-maximal inhibitory concentration (IC50) of the samples was predicted by ridge regression. The accuracy was calculated through 10-fold cross-validation based on the GDSC training dataset (15).

### Statistical Analysis

All statistical data were analyzed in the R environment (R version: 3.6.2). We applied the Wilcoxon test (Mann-Whitney test) to analyze continuous variables, whereas the Fisher’s exact test or chi-square test was used to analyze the categorical data. The survival difference was calculated using the K-M analysis methods and the log-rank test. For all statistica analyses, a P-value less than 0.05 was regarded as statistically significant.

## Results

### EMT-Related lncRNA Identification

The EMT-related genes were retrieved from the MSigDB database with the hallmark gene sets name: HALLMARK_EPITHELIAL_MESENCHYMAL_TRANSITION. As a result, a total of 200 EMT-related genes were collected (**Table S1**). We characterized the EMT-related lncRNAs through correlation analysis. According to the screening criteria, a total of 2019 EMT-related lncRNAs were identified with the absolutely Pearson coefficient > 0.3 and P-value < 0.01 (**Table S2**). The clinical information and data on lncRNA expression were merged for the downstream analysis.

### Constructing the EMT-Related lncRNA Signature

**Figure 1:**
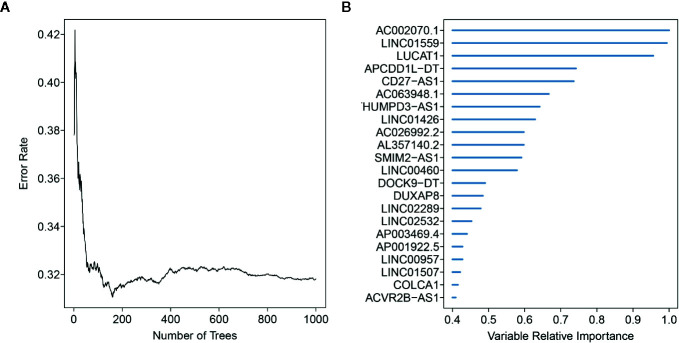
Random forest analysis for the gene selection. **(A)** The association between error rate distribution and number of trees. **(B)** Out-of-bag importance values for the genes based on the screening criterion.

Here, 512 ccRCC patients with survival time ≥ 30 days, and 2019 EMT-related lncRNAs were included in the TCGA-ccRCC cohort to identify the prognostic risk model. Through univariate Cox regression analysis, we obtained 491 lncRNAs with a significant prognostic difference. Then, the randomSurvivalForest algorithm was adopted to make a feature selection. The selected genes with a relative importance > 0.4 were further applied for multivariate stepwise Cox regression analysis. The relationship between the error rate and the number of tree is shown in **Figure 1A**, while the relative importance of genes based on the criteria is shown in **Figure 1B**. We then established an 11-lncRNA signature model by through a multivariate stepwise Cox regression analysis. The risk score for each patient in the TCGA cohort and ICGC cohort was calculated on the basis of the risk formula:

$$\begin{array}{l} Riskscore=LINC01507*(-0.035217972)+LINC00957*2(-0.049374676)\\ +LINC02532*(0.002113896)+DOCK9-DT*(-0.151293526)+AL357140.2\\ {}*(-0.649088683)+THUMPD3-AS1*(0.066728022)+AC063948.1*(0.123695894)\\ +CD27-AS1*(0.011624065)+APCDD1L-DT*(0.021343717)+LINC01559\\ {}*(0.004140941)+AC002070.1*(-0.061693196) \end{array}$$

### Prognostic Value of the EMT-Related lncRNA in ccRCC

**Figure 2:**
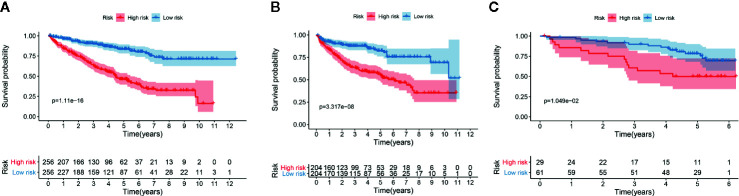
Kaplan-Meier curve analysis for the OS **(A)** and DFS **(B)** of the EMT-related signature in the TCGA dataset, and OS in the ICGC dataset **(C)**.

**Figure 3:**
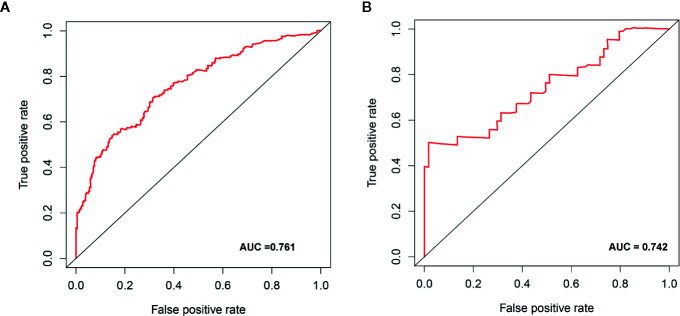
The receive operator curve (ROC) analysis for the EMT-related lncRNA signature in the TCGA dataset **(A)** and ICGC dataset **(B)**.

**Figure 4:**
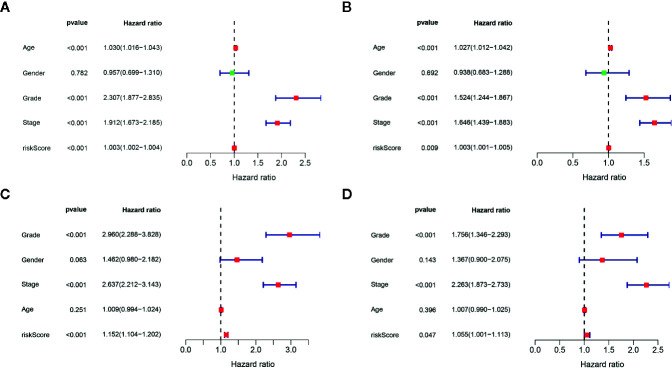
Univariate cox regression analysis and multivariate cox regression analysis of the association between EMT-related lncRNA signature and clinical factors in the OS **(A**, **B)** and DFS **(C**, **D)**, respectively.

**Figure 5:**
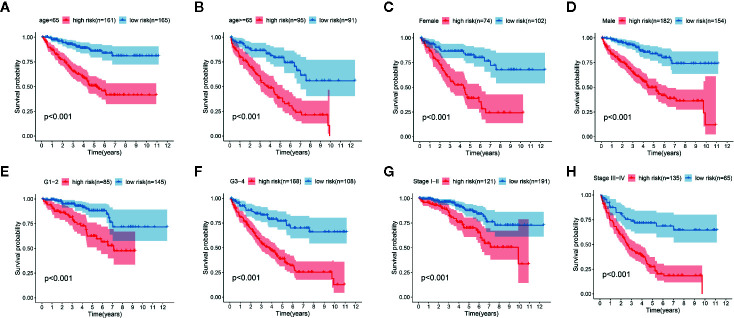
Kaplan-Meier curve analysis for the high-risk group and low-risk group stratified by clinical factors, including age **(A, B)**, gender **(C, D)**, grade **(E, F)**, stage **(G, H)**.

**Figure 6:**
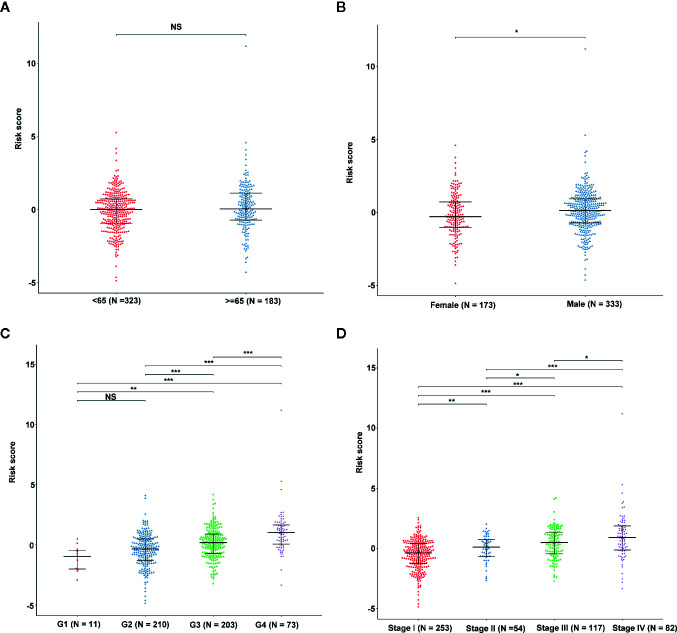
The association between EMT-related signature risk score and clinical factors, including age **(A)**, gender **(B)**, grade **(C)**, stage **(D)**. NS: Not Significant, *: P < 0.05, **: P < 0.01, ***: P < 0.001.

We determined the potential of using the EMT-related lncRNA signature to predict the overall survival (OS) and disease-free survival (DFS) to predict the prognosis of ccRCC patients. Patients were categorized into high-risk group and low-risk group based on the median risk score. Then. the Kaplan-Meier curve analysis was conducted to assess the OS and DFS outcomes between two risk groups. As depicted in **Figures 2A, B**, the OS and DFS rate of patients in the low-risk group were significantly higher than those in the high-risk group (P < 0.001). Similarly, patients in the ICGC dataset exhibited a prolonged survival time in the low-risk group when compared to the high-risk group (**Figure 2C**). Additionally, it was found the OS and DFS patients in the high-risk group were corresponded to more death cases, and high expression of APCDD1L−DT, LINC01559, AC063948.1, THUMPD3−AS1, and CD27−AS1, whereas, more alive cases and high expression level of LINC00957, LINC01507, LINC02532, AL357140.2, DOCK9−DT and AC002070.1 were reported in the low-risk group (**Figure S1**). Based on the ROC analysis results generated by the risk model, the AUC value in the TCGA cohort and ICGC cohort was reached 0.761 and 0.742, indicating a good 5-year prediction prognostic accuracy (**Figures 3A, B**). In addition, we performed a univariate cox regression and multivariate cox regression analyses to determine whether the EMT-related lncRNA signature can serve as an independent prognostic predictor for OS and DFS in ccRCC patients. As showed in **Figures 4A–D**, we observed that the lncRNA signature, stage and grade were listed as an independent predictor for OS and DFS. To further explore the prognostic value of the EMT-lncRNA signature in ccRCC patients stratified by clinical variables, we assigned the patients into different groups based on age, gender, stage and grade. Considering the different stratified analysis results, ccRCC patients in the low-risk group were characterized by a significantly prolonged OS time than patients in the high-risk group (P < 0.05) (**Figure 5**). These findings demonstrated that EMT-related lncRNA signature for OS can predict the prognosis of ccRCC without considering the impact of clinical factors. Besides, we evaluated the ability of the EMT-related lncRNAs to promote the progression of ccRCC. Of note, we found that the risk score presented a significantly increasing trend in the stage, grade (Kruskal–Wallis P < 0.05). Furthermore, the risk score in males was significant higher than in females, whereas no significant difference was observed with age (**Figure 6**). These findings suggested a higher the risk score, and a higher malignancy of the ccRCC. Therefore, the EMT-lncRNA signature could accurately predict the progression of ccRCC.

### Construction and Validation of Nomogram in the TCGA Cohort

**Figure 7:**
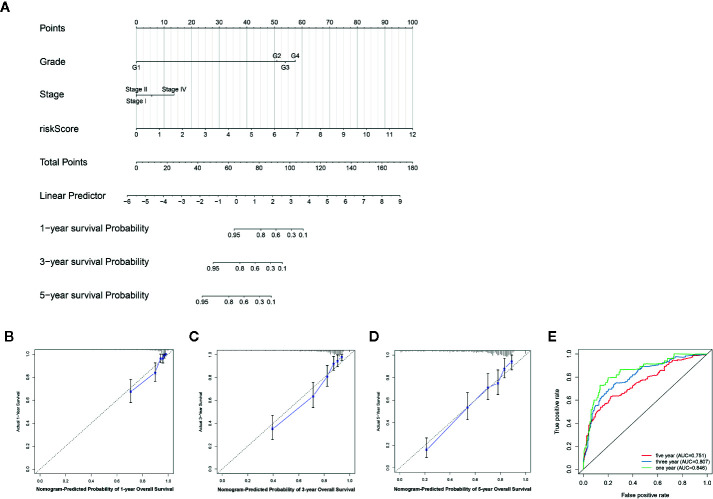
An individualized prediction model for overall survival (OS) of patients with ccRCC. **(A)** Nomogram construction for the 1-, 3- and 5- year OS prediction for the ccRCC. **(B–D)** Calibration curves analysis of the nomogram for predicting 1-, 3- and 5-year OS in the TCGA dataset. **(E)** Evaluation of the accuracy of the nomogram in 1-, 3- and 5-year by using the ROC analysis.

The nomogram was established based on the independent factors, including EMT-related lncRNA signature, stage and grade (**Figure 7A**). The calibration plots present high performance in predicting 1-year, 3-year and 5-year OS in ccRCC (**Figures 7B–D**). Furthermore, the prediction accuracy of the nomogram was evaluated via ROC analysis. Results showed that the AUC value for the 1-year, 3-year and 5-year in ccRCC was 0.846, 0.807, and 0.751, respectively (**Figure 7E**), suggesting that our nomogram was highly accurate.

### GSEA Enrichment Analysis Result of the EMT-Related lncRNA Signature

**Figure 8:**
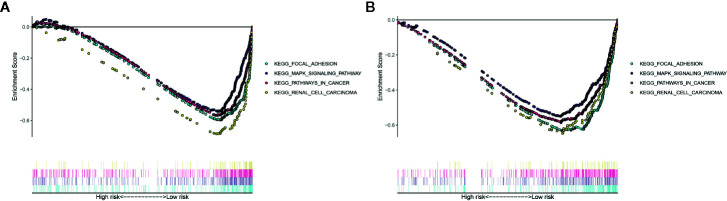
Gene Set Enrichment Analysis (GSEA) for the EMT-related lncRNA signature in the TCGA dataset **(A)** and ICGC dataset **(B)**.

The Gene Set Enrichment Analysis (GSEA) analysis was applied to identify the significant pathway associated with the high-risk group and low-risk group in the TCGA cohort and ICGC cohort. Patients in the low-risk group were mainly enriched in focal adhesion, MAPK signaling pathway, pathways in cancer and renal cell carcinoma pathway (**Figures 8A, B**). However, no significantly pathway was enriched in the high-risk group.

### Three Molecular Subtypes of ccRCC Identified by NMF Clustering

**Figure 9:**
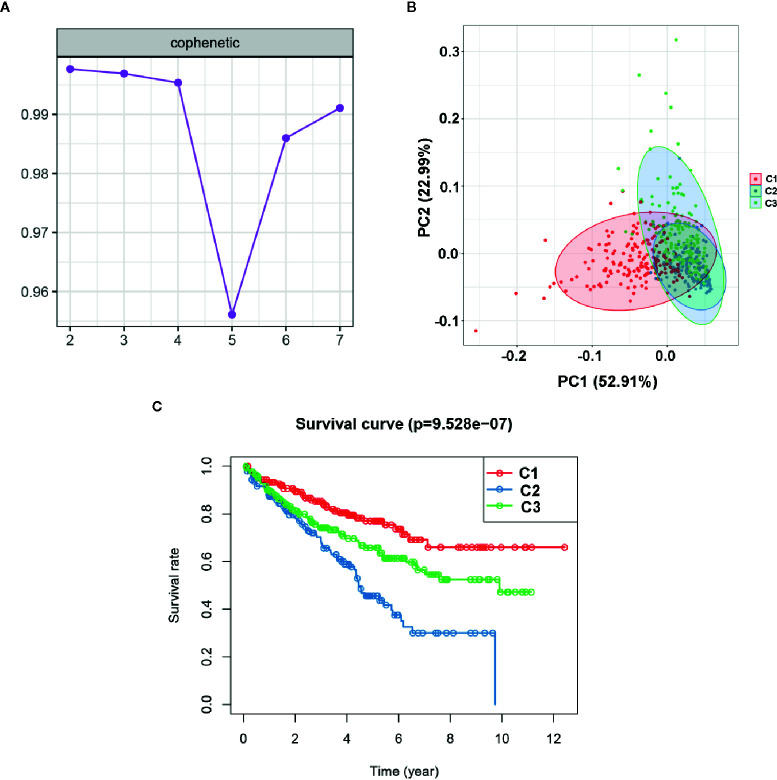
Identification of molecular subtype of ccRCC based on the EMT-related lncRNA expression in the TCGA dataset. **(A)** NMF clustering using 491 NMF-related lncRNAs. The cophenetic correlation coefficients were shown when k = 2 to k = 7. **(B)** Principal components analysis (PCA) for the EMT-related lncRNA expression. **(C)** Kaplan-Meier curve analysis for the two subtypes.

To explore the potential molecular subtypes of ccRCC based on the EMT-related lncRNAs, the lncRNAs with significant survival differences were selected following results from the univariate Cox regression analysis result. After screening, a total of 491 lncRNA with 512 patients in the TCGA cohort were included in the NMF consensus clustering analysis. The cophenetic correlation coefficients were calculated to determine the optimal k value, and k=3 was selected as the optimal k value after a comprehensively consideration (named C1, C2 and C3) (**Figure 9A**). The consensus heatmap showed a sharp and crisp boundary for each subtype, suggesting that the robustness and reliability of the subtype (**Figure S2**). Moreover, the principal component analysis (PCA) results indicated that there is a significant difference between C1, C2, and C3 (**Figure 9B**). Further, the K–M curve analysis results suggested that subtype C1 had a better overall survival than subtype C2 and C3 (P < 0.001) (**Figure 9C**). To further validate the stability of molecular subtype, we conducted an NMF clustering analysis in the ICGC cohort. Notably, similar results were obtained, except for the survival analysis results which could be attributed to the small sample size (**Figure S3**).

### Landscape of Genomic Profiling and Immune Infiltration Level in Subtypes

**Figure 10:**
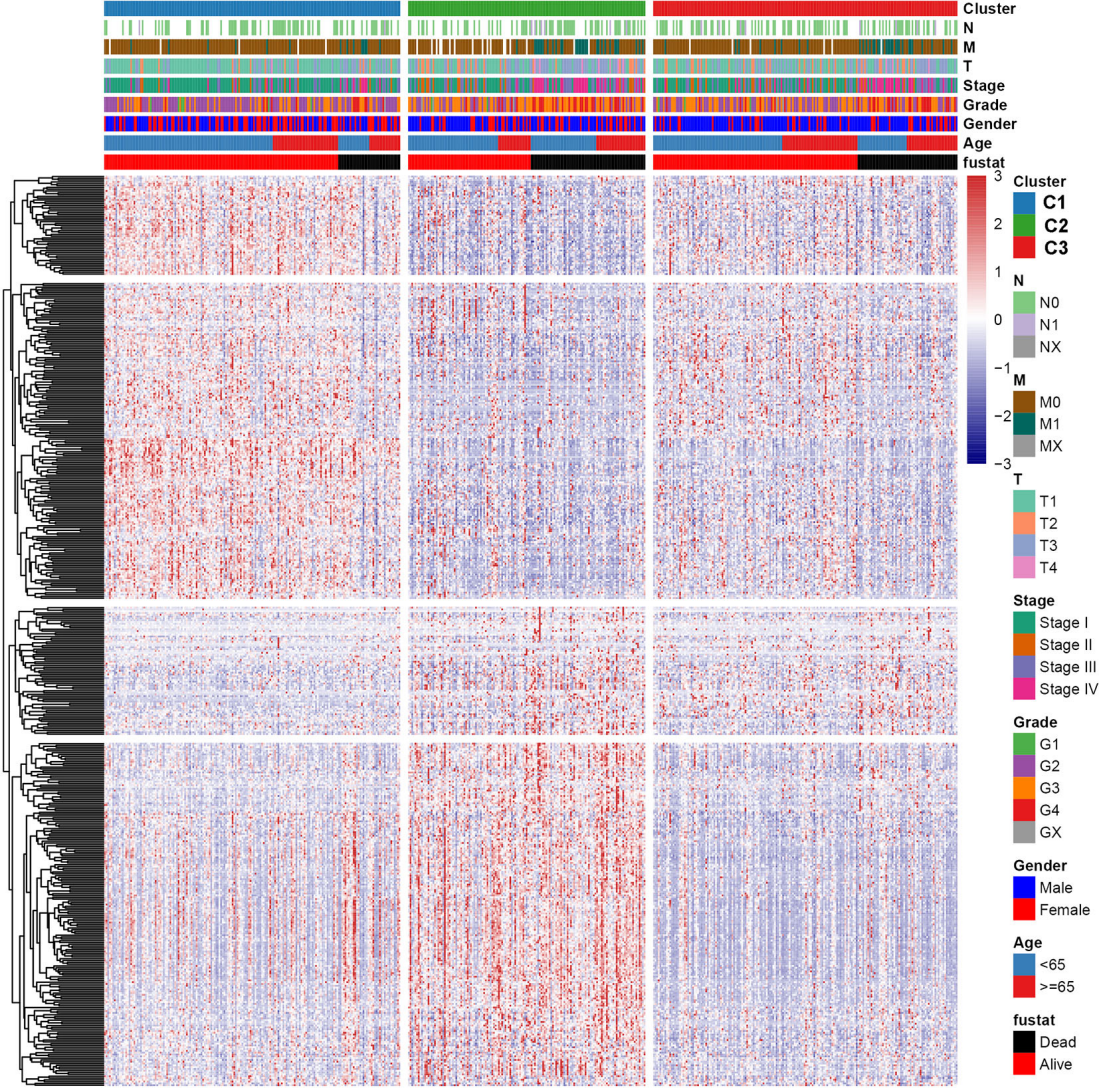
A heatmap was shown for the relationship between clinical factors, subtype and EMT-related lncRNA expression in the TCGA dataset.

**Figure 11:**
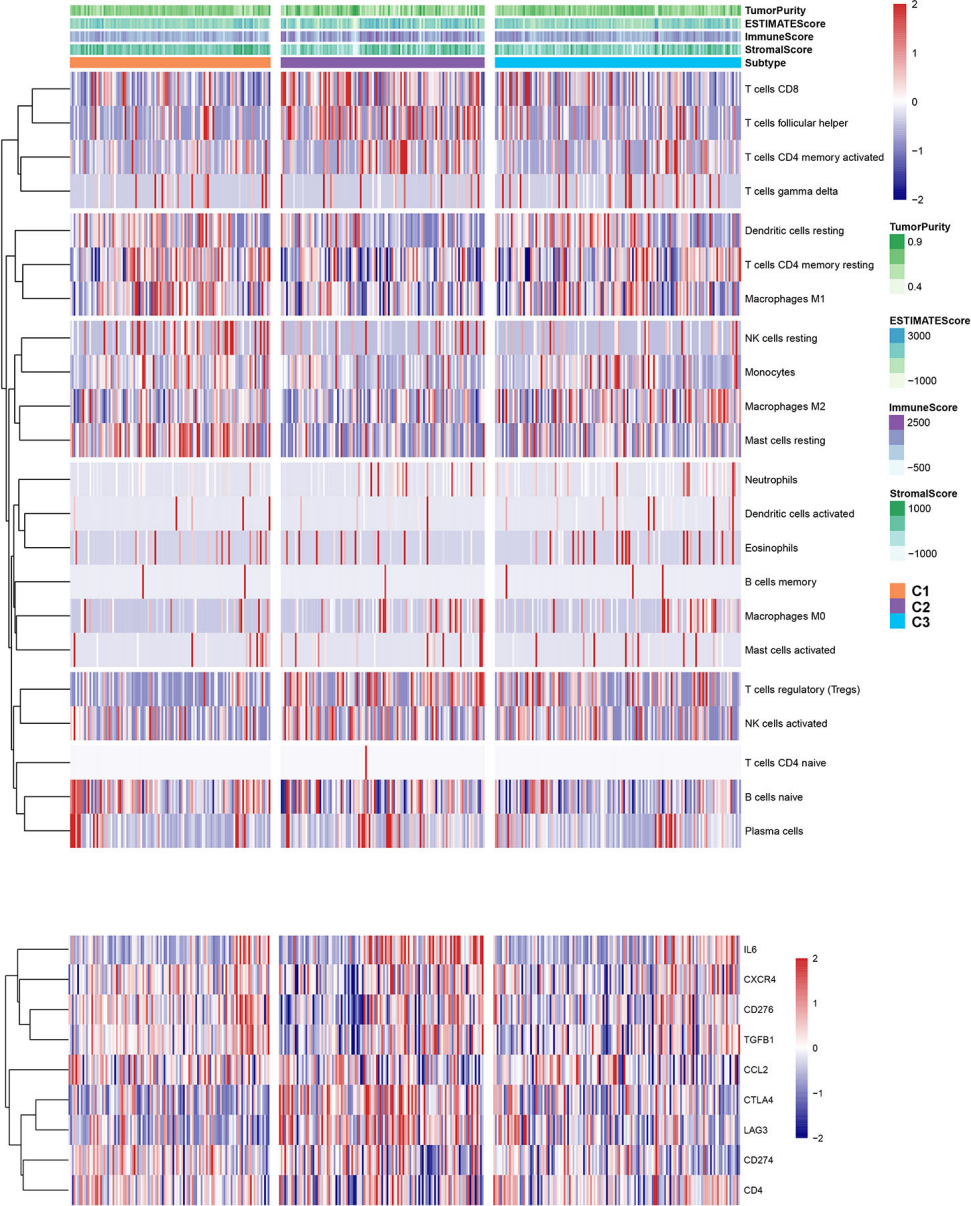
The landscape of immune cell infiltration and immune check point in the three subtypes.

To explore the relationship between subtype of ccRCC and its clinical factors in the TCGA dataset, we generated a heatmap to depict the association between the lncRNA expression, molecular subtype and clinical factors (**Figure 10**). It was found that the lncRNA expression is highly specific in subtypes, indicating that the different subtypes exhibit different functions. Moreover, we revealed that C1 was highly associated with more stage I, stage II, grade 1, and grade 2 patients, while C2 highly corresponded to more stage III, stage IV, grade 3, and grade 4 patients, demonstrating that the C2 subtype is related to the progression of ccRCC. Similarly, findings were reported in the ICGC result (**Figure S4**). Also, the VHL and PBRM1 were found to be the most common mutation genes among the subtype, which is consistent with the previously reported (**Figure S5**). Previously studies found that the EMT-related genes are associated with the tumor microenvironment, whereas they were not discovered in the ccRCC. This prompted us to further explore the relationship between molecular subtype, immune checkpoint, and immune cell infiltration level. We found that the immune cell infiltration level including CD8 T cell, follicular helper T cells, CD4 memory activated T cells and gamma delta T cells were higher in C2 when compared to C1 and C3, whereas high levels of resting Dendritic cells, CD4 memory resting T cells and M1 Macrophages were found in subtype C1 (**Figure 11**). In addition, the overall expression levels of known immune checkpoints including IL6, CXCR4, CD276, TGFB1, CCL2, CTLA4, LAG3, CD274, and CD 4 were relatively higher in subtype C2 compared to subtype C1 and subtype C3 (**Figure 11**).

### More Sensitivity to Immuno/Chemotherapies for C2 Subtype

**Figure 12:**
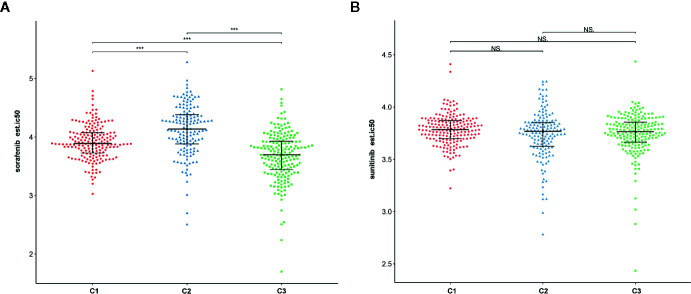
The relationship between chemo drug and three subtypes. **(A)** Sorafenib. **(B)** Sunitinib. NS: Not Significant, ***: P < 0.001.

To assess the likelihood of how the three subtypes respond to immunotherapy, we used the TIDE algorithm. Results demonstrated that subtype C2 (30.3%, 44/145) was more likely to respond to immunotherapy than C3 (28.0%, 52/186) and C1 (16.0%, 29/181) (Kruskal–Wallis P < 0.001). In addition, sorafenib and sunitinib chemo drugs were applied to the treatment of metastatic RCC cases. Therefore, we further evaluated the response of three subtypes to two chemo drugs using the GDSC cell line data set. As shown in **Figures 12A, B**, it was evident that subtype C2 was more sensitive to sorafenib chemo drug compared to subtype C1 and C3. However, no significant difference was observed in the sunitinib drugs among the subtypes. Notably, subtype C2 was associated with more advanced patients and was susceptible to local recurrence or distant metastasis. In a nutshell, we concluded that the C2 subtype can benefit from the sorafenib therapy.

## Discussion

ccRCC is the most frequently subtype of renal cancer, which is highly associated with poor prognosis and distant metastasis (16). Exploring potential biomarkers is vital to the management and prognosis of ccRCC. Accumulated evidence shows that EMT is associated with the progression and metastasis of cancer (17). However, a majority of the studies focused on the impact of the EMT in tumor development and treatment, whereas fewer studies addressed the prognostic value of EMT-related genes or lncRNAs in cancer, especially in ccRCC.

In the present study, we constructed a novel and efficient EMT-related lncRNA signature based on the TCGA dataset and validated its efficiency in the ICGC dataset. The ROC analysis result in the TAGC dataset and ICGC dataset confirmed the high prognostic value of our signature. Moreover, the signature showed a significant correlation with clinical factors, further supporting the robustness of its prognostic value. We also demonstrated that the EMT-related lncRNAs can potentially be utilized as an independent predictor for the OS and DFS in the TCGA dataset. The nomogram constructed by the stage, grade and our signature showed a high performance in the 1-, 3- and 5-year, which may contribute to promote the individualized treatment of ccRCC patients. Collectively, these findings demonstrated that a great prognostic value of our EMT-related lncRNA signature, thus may provide a theoretical basis for EMT-related targeted therapies. In addition, our GSEA results indicated that numerous pathways were significantly enriched in the low-risk group, suggesting that EMT exerts more regulatory roles in the low-risk group compared to the high-risk group.

Furthermore, most of the lncRNAs in our signature had been reported in the cancer types. For instance, LINC00957 was proved to be a potential biomarker and therapeutic target for colorectal cancer patients (18); LINC02532 was demonstrated to act as an oncogene in gastric cancer (GC) and promoted the GC cell proliferation, migration, and invasion (19); DOCK9-DT was revealed to play a protective role in the prognosis of thyroid carcinoma (20); THUMPD3-AS1 regulated non-small cell lung cancer cell self-renewal via the expression of miR-543 and ONECUT2, and THUMPD3-AS1 can serve as a potential biomarker or therapeutic target in non-small cell lung cancer (21). Additionally, APCDD1L-DT exhibits a significant prognostic value in lung squamous cell carcinoma (LUSC) and can independently predict the overall survival in LUSC patients (22). LINC01559, which can promote the pancreatic cancer (PC) cell proliferation and migration via the YAP-mediated pathway, providing a novel target therapy for clinical treatment of patients with PC (23). Moreover, the LINC01559 can act as an potential oncogene, thereby accelerating the resistance of hepatocellular carcinoma to oxaliplatin by sponging miR-6783-3p (24). Currently, the molecular mechanism or prognostic value of other lncRNAs including LINC01507, AL357140.2, AC063948.1, CD27-AS1, and AC002070.1 are elusive. Despite important prognostic value of lncRNAs, future experiments are necessary to elucidate their role in ccRCC.

Although numerous ccRCC molecular subtypes based on gene expression have been proposed in recent years, those associated with the EMT-related lncRNAs are yet to be fully explored. Herein, we assessed the ccRCC subtypes associated with EMT, using the NMF algorithm to perform a consensus clustering to the ccRCC samples based on EMT-related lncRNA. Of note, three molecular subtypes (C1, C2 and C3) were identified and validated in the TCGA and ICGC datasets. The PCA findings confirmed the robustness and reliability of the subtypes identified in the present study. Furthermore, the K–M curve result suggested that subtype C1 was was associated with better survival outcomes and in a majority of early-stage patients, whereas subtype C2 was associated with worse survival outcomes and in more advanced-stage patients. Previous investigations reported that tumor microenvironment is associated with EMT. Thus, the relationship between tumor microenvironment and subtype were further explored. In the present work, we found that the CD8 + T cell, follicular helper T cells, CD4 memory activated T cells and gamma delta T cells were presented high expression level in subtype C2, interestingly, the overall expression level of immune checkpoints also showed a high expression level in subtype C2. These results can be interpreted that the anti-tumor effect of high level of T cell infiltration is offset by the strong immunosuppressive pathway activated by over-expressed immune checkpoint proteins, and might provide the likelihood of immunotherapy for C2 patients (25, 26). We further evaluated the likelihood of response to immunotherapy in the three subtypes using the TIDE algorithm. Notably, subtype C2 was found to be more likely to respond to immunotherapy. In addition, we demonstrated that sorafenib was more sensitive to the subtype C2. The sorafenib has been used in the treatment of metastatic RCC cases, with considerable progress (27). Since the subtype C2 corresponded to more advanced stage patients, therefore, we inferred that subtype C2 could more likely be utilized for treatment by the sorafenib.

This study has a few limitations: Firstly, our risk signature was constructed based on the public dataset, some important clinical information was not complete and unavailable for analysis, this could introduce potential basis or errors. Secondly, the ICGC dataset sample size was small compared to the TCGA dataset, and may have contributed to the non-significant results, such as in three molecular subtype survival analysis. Thirdly, the EMT-related lncRNA signature should be validated in future studies through in vivo and in vitro experiments.

In conclusion, the present work developed and validated an EMT-related lncRNA signature that can be utilized as a reliable tool for predicting individualized prognosis and for decision-making when treating ccRCC patients. In addition, three molecular subtypes were revealed, which may contribute to understanding the molecular mechanism of ccRCC and provide references for clinicians to develop an individualized treatment for ccRCC patients.

## Data Availability Statement

Publicly available datasets were analyzed in this study. The datasets generated for this study can be found in https://portal.gdc.cancer.gov/ and https://dcc.icgc.org.

## Author Contributions

YL and JH designed the study. CH and FZ collected the clinical information and gene expression data. WZ analyzed the data and wrote the manuscript. YL and JH revised the manuscript. All authors contributed to the article and approved the submitted version.

## Funding

This study was supported by the Health Science Research Personnel Training Program of Fujian Province (2017-CXB-22), Fujian Science and Technology Plan Guiding Projects (2019D026) and Xiamen Medical Advantage Subspecialty Vascular Access Construction Fund ([2018] 296). Moreover, we also thank the support of the research program fund of the Fifth Hospital of Xiamen.

## Conflict of Interest

The authors declare that the research was conducted in the absence of any commercial or financial relationships that could be construed as a potential conflict of interest.

## Supplementary Material

Supplementary Figure 1 The EMT-related lncRNA signature risk score analysis of the OS **(A)** and DFS **(B)** in the TCGA dataset. The upper panel represent the lncRNA risk score distribution, medium panel represent the cases distribution and the lower panel represent the the expression level of each prognostic lncRNA.

Supplementary Figure 2 The NMF consensus heatmap of the three subtypes in the TCGA dataset.

Supplementary Figure 3 Identification of molecular subtype of ccRCC based on the EMT-related lncRNA expression in the ICGC dataset. **(A)** NMF clustering using 491 NMF-related lncRNAs. The cophenetic correlation coefficients were shown when k = 2 to k = 7. **(B)** Principal components analysis (PCA) for the EMT-related lncRNA expression. **(C)** The NMF consensus heatmap of the three subtypes.

Supplementary Figure 4 A heatmap was shown for the relationship between clinical factors, subtype and EMT-related lncRNA expression in the ICGC dataset.

Supplementary Figure 5 The oncoPrint was constructed by the three molecular subtypes, individual patients represented in each column.
