# Supplementary figures and images for: Identification of Epithelial–Mesenchymal Transition-Related lncRNA With Prognosis and Molecular Subtypes in Clear Cell Renal Cell Carcinoma
Source: Front Oncol. 2020 Nov 25;10:591254. doi: 10.3389/fonc.2020.591254 (PMC7724112; doi:10.3389/fonc.2020.591254)

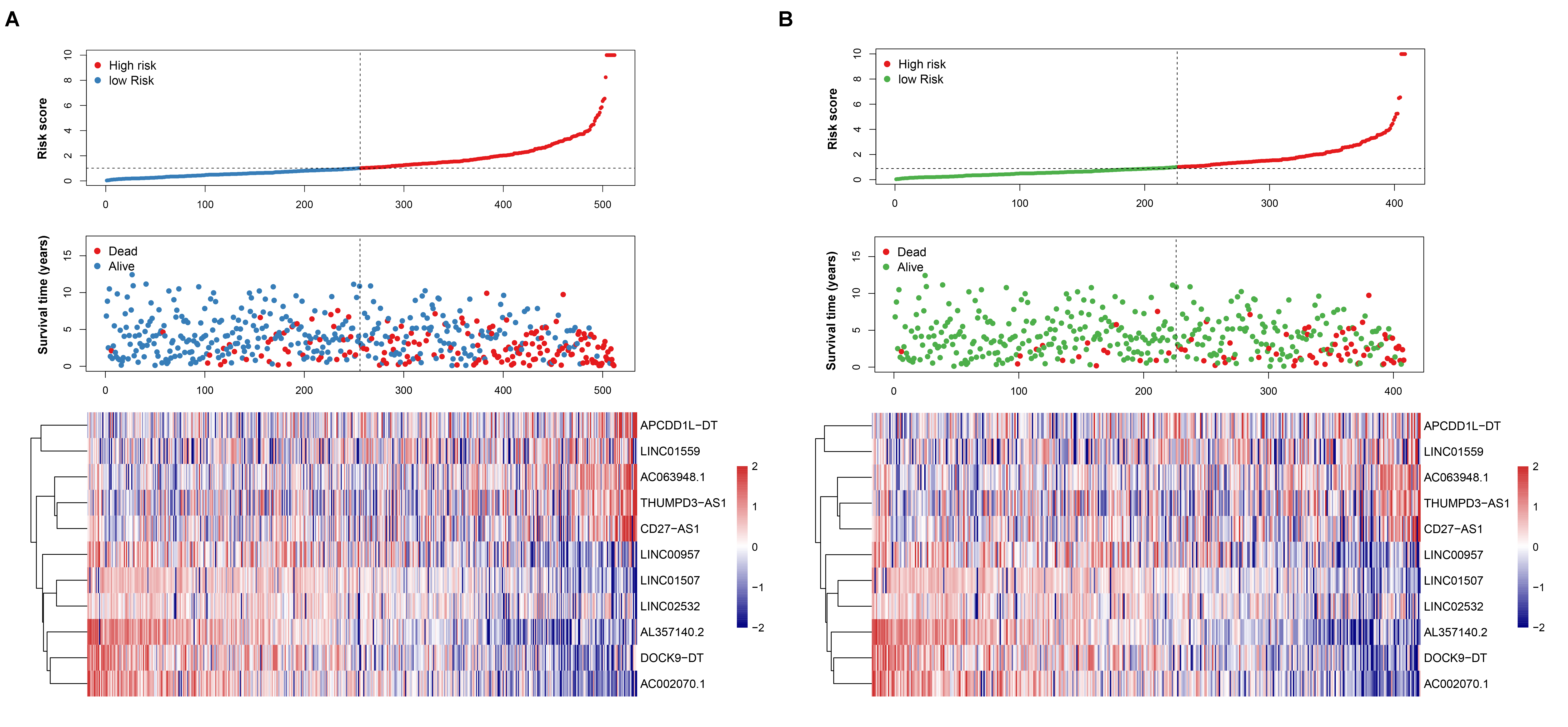

Supplement: Supplementary Figure 1 — The EMT-related lncRNA signature risk score analysis of the OS (A) and DFS (B) in the TCGA dataset. The upper panel represent the lncRNA risk score distribution, medium panel represent the cases distribution and the lower panel represent the the expression level of each prognostic lncRNA. [file Image_1.jpeg]

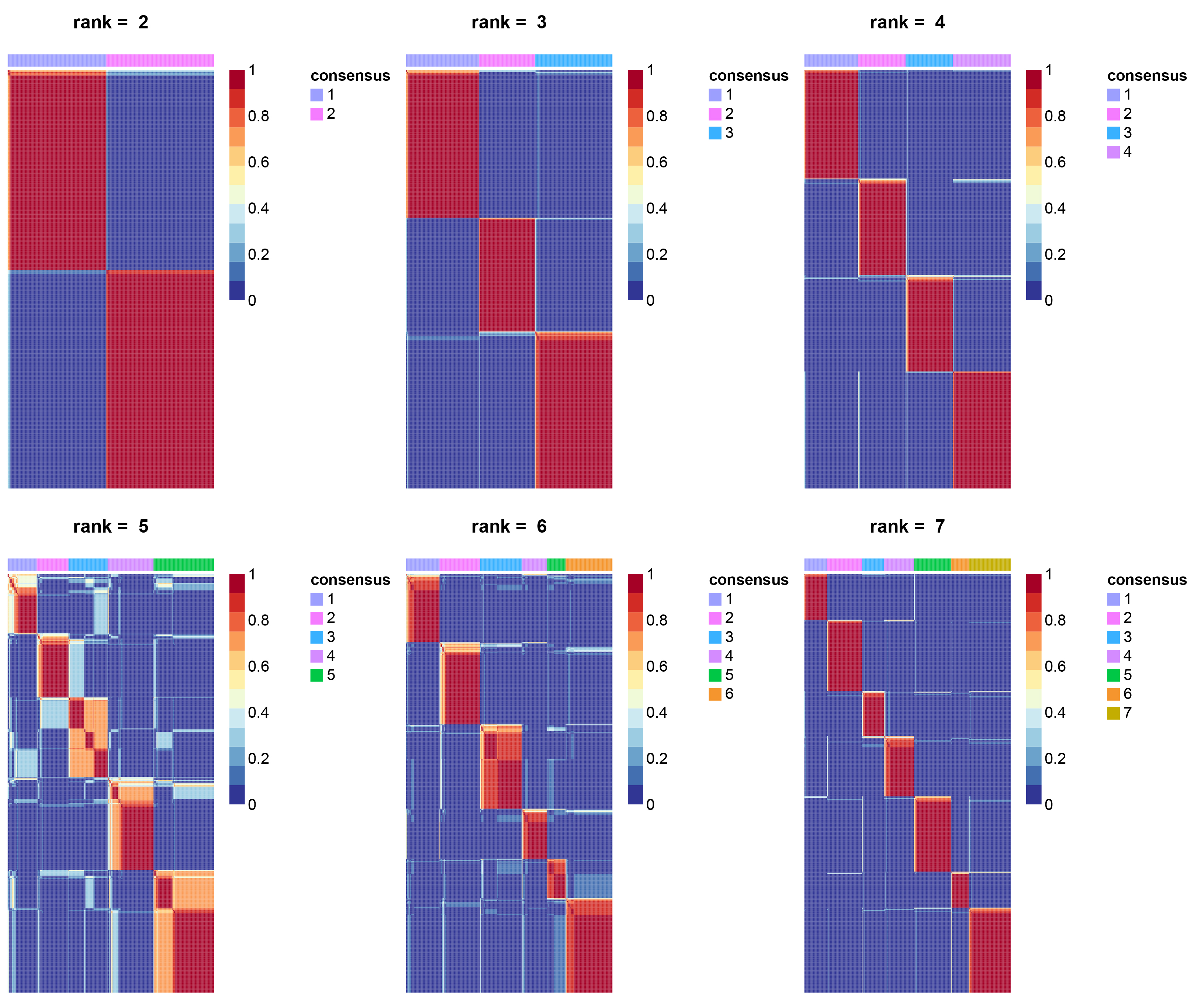

Supplement: Supplementary Figure 2 — The NMF consensus heatmap of the three subtypes in the TCGA dataset. [file Image_2.jpeg]

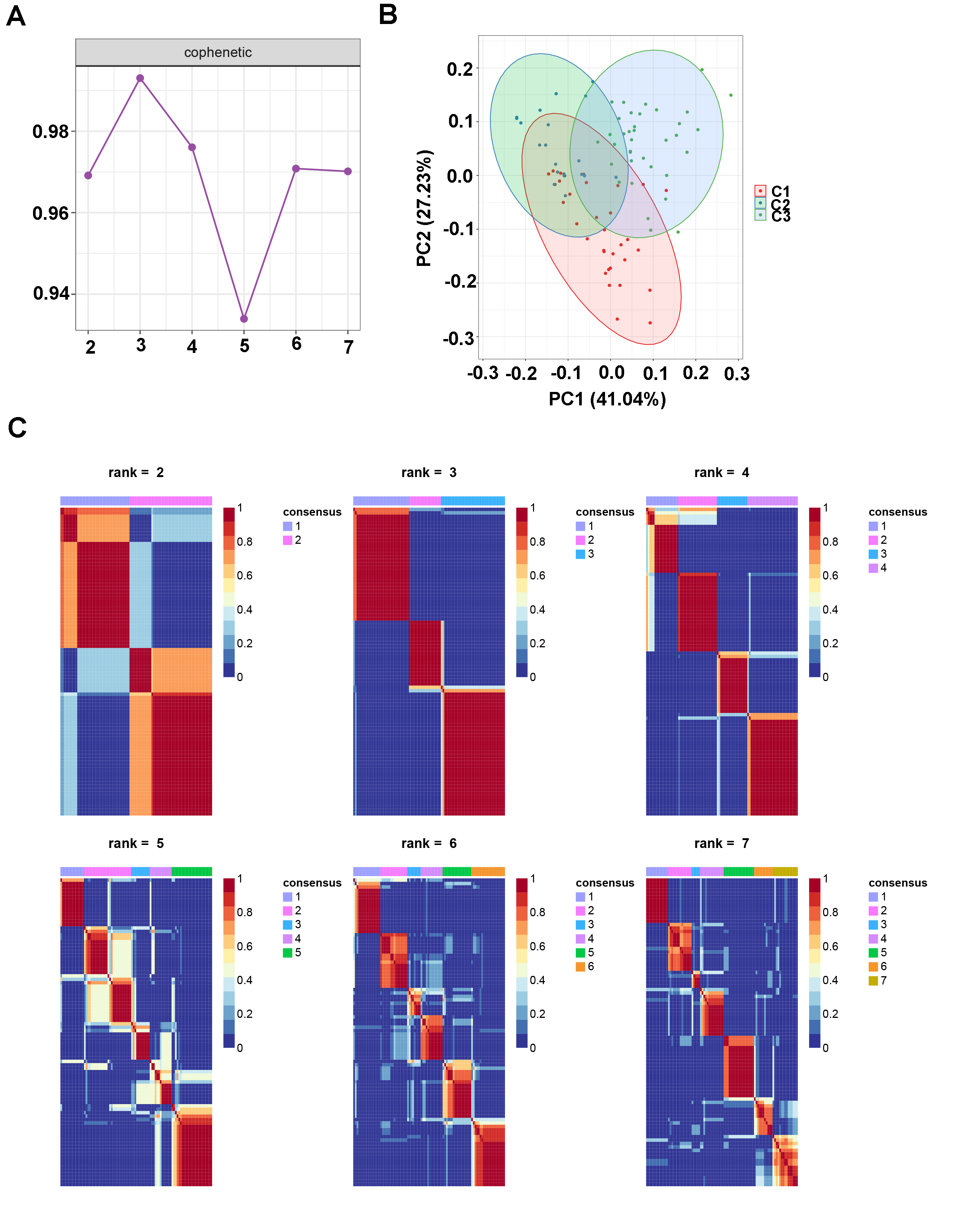

Supplement: Supplementary Figure 3 — Identification of molecular subtype of ccRCC based on the EMT-related lncRNA expression in the ICGC dataset. (A) NMF clustering using 491 NMF-related lncRNAs. The cophenetic correlation coefficients were shown when k = 2 to k = 7. (B) Principal components analysis (PCA) for the EMT-related lncRNA expression. (C) The NMF consensus heatmap of the three subtypes. [file Image_3.jpeg]

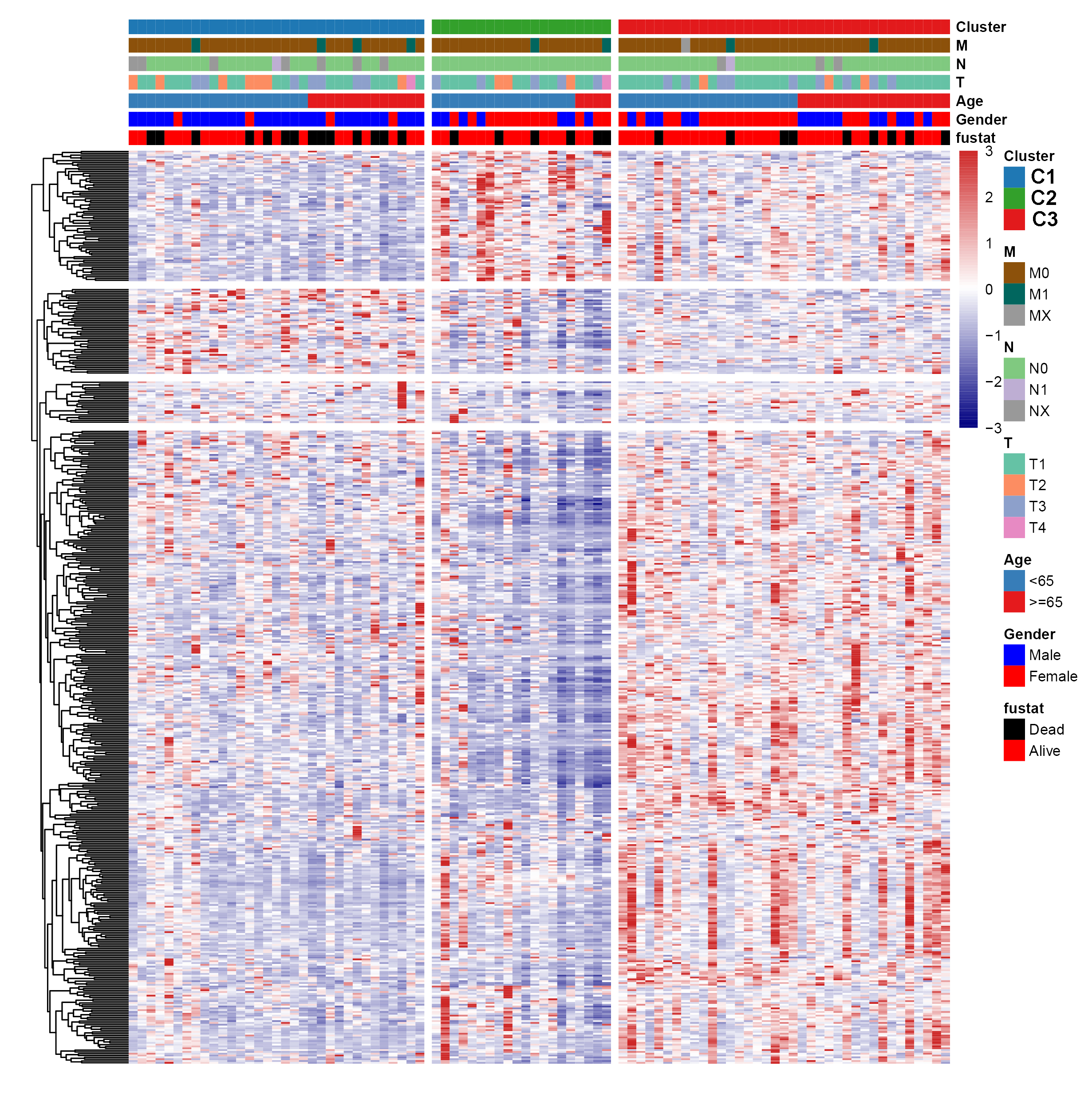

Supplement: Supplementary Figure 4 — A heatmap was shown for the relationship between clinical factors, subtype and EMT-related lncRNA expression in the ICGC dataset. [file Image_4.jpeg]

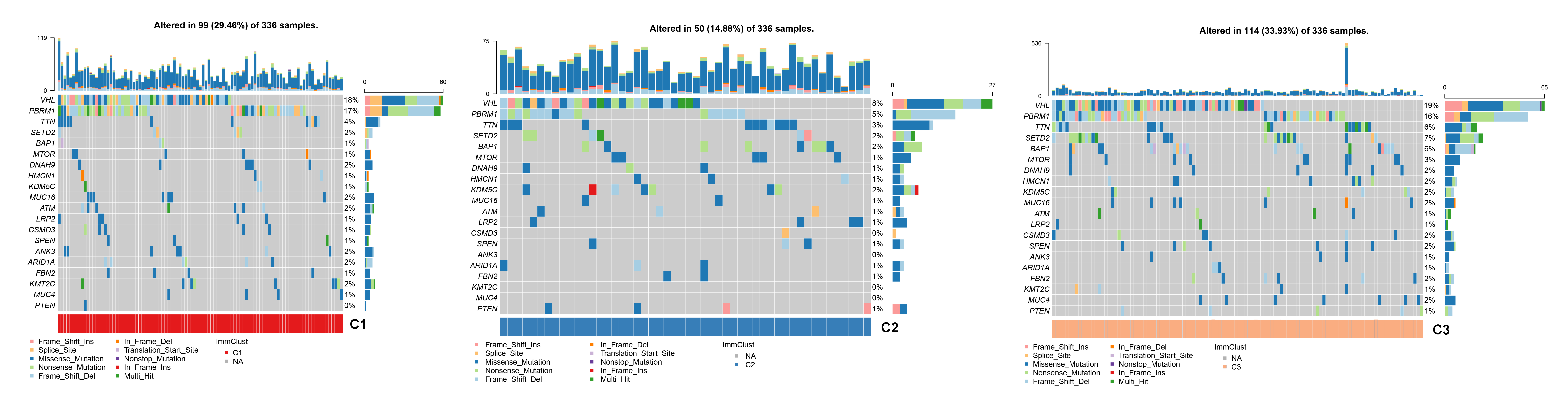

Supplement: Supplementary Figure 5 — The oncoPrint was constructed by the three molecular subtypes, individual patients represented in each column. [file Image_5.jpeg]
